# The Role of Slow Speech Amplitude Envelope for Speech Processing and Reading Development

**DOI:** 10.3389/fpsyg.2017.01497

**Published:** 2017-08-31

**Authors:** Paula Ríos-López, Monika T. Molnar, Mikel Lizarazu, Marie Lallier

**Affiliations:** ^1^Developmental Language Disorders, Basque Center on Cognition, Brain and Language Donostia-San Sebastián, Spain; ^2^Laboratoire de Sciences Cognitives et Psycholinguistique, Centre National de la Recherche Scientifique, Ecole Normale Supérieure Paris, France

**Keywords:** reading, amplitude envelope, phonological processing, speech-in-noise, auditory entrainment, language development

## Abstract

This study examined the putative link between the entrainment to the slow rhythmic structure of speech, speech intelligibility and reading by means of a behavioral paradigm. Two groups of 20 children (Grades 2 and 5) were asked to recall a pseudoword embedded in sentences presented either in quiet or noisy listening conditions. Half of the sentences were primed with their syllabic and prosodic amplitude envelope to determine whether a boost in auditory entrainment to these speech features enhanced pseudoword intelligibility. Priming improved pseudoword recall performance only for the older children both in a quiet and a noisy listening environment, and such benefit from the prime correlated with reading skills and pseudoword recall. Our results support the role of syllabic and prosodic tracking of speech in reading development.

## Introduction

The amplitude envelope of speech contains crucial information about prosodic and stress patterns, speech rate, tonal contrasts and intonational information necessary for successful speech perception. Specifically, multiple studies have shown that the slow components of the speech amplitude envelope (below 8 Hz) – that code for prosodic and syllabic information (Houtgast and Steeneken, 1985) – contribute significantly to the perception of the rhythmic structure of speech (Rosen, 1992; Hickok and Poeppel, 2007; Abrams et al., 2008; Giraud and Poeppel, 2012).

Pioneering behavioral evidence on the role of the slow temporal fluctuations of speech for successful speech perception has received notable neurophysiological support during the last decade. In particular, numerous studies have shown that the oscillatory activity in the brain “phase-locks” to the temporal structure of auditory speech stimuli (Suppes et al., 1997, 1998; Ahissar et al., 2001; Abrams et al., 2008; Aiken and Picton, 2008; Nourski et al., 2009; Peelle and Davis, 2012; Bourguignon et al., 2013; Peelle et al., 2013; Molinaro et al., 2016). One of the reasons why experiments have proliferated in this field concerns the fact that endogenous brain oscillatory activity which coincides with typical frequencies of salient speech features (i.e., gamma, theta and delta, with phonemes, syllables, and stress, respectively) could represent a good candidate mechanism that would support speech parsing (Ghitza, 2012; Poeppel, 2014; Leong and Goswami, 2015): phonemes appear at rates of approximately 80 ms (gamma band, >25 Hz), syllables at approximately 200 ms (theta band, 4–8 Hz), and stressed syllables at around 500 ms (delta band <4Hz; Ghitza and Greenberg, 2009; Goswami, 2011). Based on this phenomenon, several studies have described the properties of neural oscillatory mechanisms in relation to the human ability to encode and segment speech (e.g., Poeppel, 2003; Giraud et al., 2007; Goswami, 2011; Giraud and Poeppel, 2012; Gross et al., 2013). For example, according to the “asymmetric sampling in time” hypothesis (Poeppel, 2003; Giraud and Poeppel, 2012), tracking the speech amplitude envelope at prosodic and syllabic rates would allow the brain to rely on some acoustic landmarks on which to anchor “neural entrainment,” i.e., synchronization of the brain oscillatory activity to external (e.g., auditory) signals. Complementary hypotheses suggest that neural oscillatory mechanisms would allow neuronal responses to be amplified and highly excitable when critical phonological, prosodic and syllabic events occur in speech streams (Lakatos et al., 2008; Schroeder and Lakatos, 2009).

Supporting these hypotheses, several experiments found a positive correlation between speech intelligibility and neural entrainment to the prosodic and syllabic properties of the auditory speech stimuli. Relevant to the current study, speech comprehension is accompanied by enhanced phased-locking values to slow amplitude speech modulations in the auditory cortex (Peelle and Davis, 2012; Peelle et al., 2013). The importance of perceiving the structure of the slow amplitude envelope of speech may be even more significant when speech intelligibility is challenged by competing background noises (which is the most common everyday listening situation). Accordingly, the role of such an amplitude-tracking mechanism during speech perception in noise has been investigated by several studies (Zion Golumbic et al., 2012, 2013; Ding and Simon, 2014). Specifically, studies on selective attention suggest that neural phase locking to the slow speech amplitude envelope of an auditory attended stimulus may help to ignore other simultaneous auditory stimuli by enhancing the selection of information to be attended and reducing the allocation of attentional resources to information that needs to be ignored (Schroeder and Lakatos, 2009; Kerlin et al., 2010; Ding and Simon, 2012; Zion Golumbic et al., 2012).

In a different line of research, perception of the slow amplitude envelope of speech has also been linked with reading abilities. Ample evidence supports the idea that atypical perception of the amplitude envelope of speech might lead to an abnormal acquisition of phonological representations, which would in turn lead to reading difficulties such as developmental dyslexia (Goswami et al., 2002, 2010). For example, Goswami et al. (2010) showed that processing of amplitude envelope structure, —specifically rise time sensibility— predicted phonological awareness skills, as measured via a rhyming task.

In line with the hypotheses on the role of oscillatory mechanisms for speech perception mentioned above, it has been proposed that phonological disorders underlying reading difficulties could stem from an atypical auditory sampling of temporal speech features (e.g., Goswami, 2011; Lehongre et al., 2013). The “temporal sampling framework” hypothesis suggests that reading difficulties in dyslexic individuals would stem from an atypical (asynchronous) tracking of the speech amplitude envelope corresponding to prosodic information, such as syllabic stress, in particular (Goswami, 2011; Goswami and Leong, 2013). In this framework, atypical temporal sampling of syllabic stress is thought to hamper the development of phonological skills, and in turn, reading acquisition. Accordingly, there is consistent evidence showing impaired synchronization to the speech amplitude envelope at low frequencies in dyslexic children and adults (Hämäläinen et al., 2005, 2012; Corriveau et al., 2007; Abrams et al., 2009; Goswami et al., 2010; Power et al., 2013). Hämäläinen et al. (2012) showed that dyslexic adults exhibited weaker neural phase locking to noises modulated in amplitude at 2 Hz compared to controls. The results of Hämäläinen et al. (2012) suggest that normal reading acquisition may depend on the adequate neural and attentional synchronization to prosodic and syllabic speech events. Interestingly, the temporal sampling framework, Goswami (2011; Goswami et al., 2014) can explain why difficulties in integrating speech information at low rates could also cause problems to integrate speech units that oscillate at higher frequencies, such as phonemes. This is a critical point in Goswami’s theory, since it offers an explanation why impaired representations and manipulation of phonemes strongly predict reading disorders across alphabetic languages (Hulme et al., 2005; Ziegler et al., 2010; Caravolas et al., 2012). Neural oscillations at low and high frequency bands are not independent. In particular, cortical oscillations reflect a “nesting” behavior such that the power of the brain responses in high frequency bands couples to the phase of lower frequencies generating a coupling (synchrony) between various neural oscillatory rates (Lakatos et al., 2005; Canolty et al., 2006; Gross et al., 2013). In other words, a precise temporal allocation of attention over prosodic and syllabic temporal speech features (i.e., subtended by neural entrainment) would enhance attentional focusing on phonemic information that falls within the attentional window (Lallier et al., 2017). Hence, the nesting of neural oscillators can explain why fast phonemic integration might depend on the efficient coding of slower speech rates (Ghitza, 2011; Giraud and Poeppel, 2012).

In addition to auditory oscillatory processing deficits, children and adults with reading difficulties also exhibit pronounced difficulties in speech-in-noise tasks (Wible et al., 2002; Bradlow et al., 2003; Ziegler et al., 2009; Boets et al., 2011; cf. Hazan et al., 2009). Such difficulties in dyslexia may arise from impoverished intelligibility of phonemic information since under noise circumstances, the saturation of the signal at the phonemic level is high and listeners cannot identify phonemic features as efficiently as in quiet conditions (i.e., “informational masking” in speech-in-noise, Brungart et al., 2001). Hence, it is possible that speech-in-noise difficulties associated with reading deficits also result from impaired auditory entrainment to speech amplitude envelope at prosodic and syllabic slow rates.

### The Current Study

Taking into account the above-described evidence on the relation between slow-amplitude speech envelope processing and spoken language intelligibility, in addition to the putative link of these aspects with reading, the aim of this study was to test the link between auditory entrainment to speech, understood as the ability to synchronize to the (quasi)rhythmic modulations in the speech signal, and reading skills in typically developing children through a behavioral paradigm.

**FIGURE 1:**
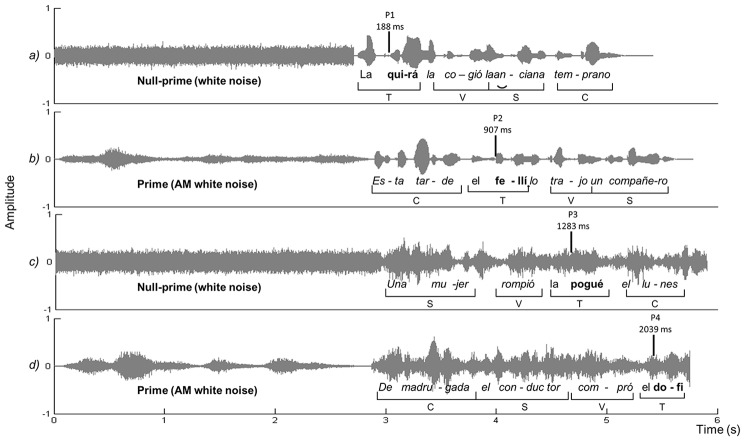
This figure shows the audio signal of four experimental sentences in the temporal domain, with time in seconds in the *x*-axis and amplitude in the *y*-axis. Because four different experimental sentences are presented, slight sentence duration differences are present. The mid-left part of the graph shows the prime (Null-prime vs. Prime) and the mid-right part the experimental sentence (including the target pseudoword). Target pseudowords are highlighted in bold. Sentence(a) corresponds to the Quiet null-prime condition; sentence (b) is an example of the Quiet prime condition. Sentences (c) and (d) correspond to the Noise listening condition (six-talker babble) in the Null-prime condition [sentence (c)] and in the Prime condition [sentence (d)]. AM, amplitude modulated.

In this experiment, children of Grades 2 and 5 listened to Spanish sentences presented in either quiet or noisy listening conditions, before they had to recall an embedded target pseudoword from the sentences (see **Figure 1** for a graphical depiction of the paradigm). Importantly, each sentence was primed with its own slow amplitude envelope that was constructed of white noise modulated with the given sentence frequencies below 8 Hz (adapted from Peelle and Davis, 2012; for further details on the stimuli, see the Materials and Methods section). In the experiment, first the children heard the slow amplitude envelope of the target sentence as prime (that contained no phonemic information, only prosodic and rhythmic information), and then the children heard the target sentence natural production (that contained all the acoustic-linguistic information of speech, including the phonetic and prosodic information). This way, only the sentence slow amplitude envelope was repeated across the prime and the target sentence.

The aim of this design was to test whether it is possible to boost phonemic encoding and the target pseudoword intelligibility in the target sentence by enhancing auditory entrainment to the slow rhythmic structure of the sentence (through priming and/or repeating the sentence amplitude envelope). Because the auditory cortical activity couples with the temporal envelope of speech (Suppes et al., 1997, 1998; Ahissar et al., 2001; Abrams et al., 2008; Aiken and Picton, 2008; Nourski et al., 2009; Peelle and Davis, 2012; Bourguignon et al., 2013; Peelle et al., 2013; Molinaro et al., 2016), we predicted the sentence amplitude envelope prime to elicit auditory entrainment to the (quasi)rhythmic properties of the sentence before listening to the spectrally complete sentence. Specifically, we predicted that such preparation/priming would enhance the processing of the same subsequent rhythmic structure, and, in turn, its intelligibility through better phonemic encoding. Indeed, recent research in the field of cross-domain effects of music on speech has shown that priming speech with music rhythms that match speech prosodic features improves phonological processing, as measured via pseudoword recall (Cason and Schön, 2012), and that rhythmic priming also contributes to better performance in other language related tasks, such as in a grammaticality judgment task (Przybylski et al., 2013).

We decided to use the low-pass filtered (<8 Hz) amplitude envelope of the sentences rather than a periodic rhythmic stimulation at a specific frequency for two main reasons: (1) by priming the target sentence with its slow amplitude envelope below 8 Hz, we were able to prime both the prosodic and syllabic rhythmic structures of the subsequent sentence in order to maximize the potential beneficial effect of the prime; (2) because we know that entrainment is triggered by statistical regularities different from periodic signals (Obleser et al., 2012; Goswami and Leong, 2013), such as strong-weak syllable stress patterns (Rothermich et al., 2012), we considered that the aperiodic statistical regularities such as the ones contained in our prime would be good candidates to elicit entrainment.

If slow speech amplitude envelope tracking is critical for speech intelligibility, priming the sentence with its slow amplitude envelope (<8 Hz) should foster auditory entrainment to both prosodic (0–4 Hz) and syllabic (4–8 Hz) speech rhythms, and improve pseudoword recall (Lallier et al., 2017).

We also presented the target sentences in noise. We predicted that if speech amplitude envelope tracking at prosodic and syllabic rates plays an essential role in the segregation of a target signal from a noisy environment, the primes should also increase the target sentence intelligibility presented in babble noise (“Cocktail Party effect”; Cherry, 1953). Since the speech amplitude envelope at prosodic, syllabic and phonemic rates is strongly masked by this type of noise (Brungart et al., 2001), we expected performance to be considerably impoverished in the null-prime condition, as opposed to in the prime condition. It was assumed that priming noisy sentences with their prosodic- and syllabic-rate structure would enhance auditory entrainment to the rhythmic structure of the sentence helping children to (selectively) attend to prosodic and syllabic amplitude fluctuations in the target sentence and facilitating in turn phonemic processing at a faster rate. In the prime condition, pseudoword recall performance was therefore expected to improve compared to the null-prime condition.

Based on current theories on reading disorders and dyslexia (Goswami, 2011), we finally hypothesized that children with low reading skills would be less sensitive to the slow speech amplitude envelope in null-primed sentences. Therefore, we expected low reading skills to be linked to larger benefit from the speech amplitude envelope prime on their pseudoword recall. Indeed, children with good reading skills should be overall more sensitive than children with low reading skills to prosodic and syllabic speech features and would not be greatly helped from the prime.

In the line of the present work, several previous studies have tested the predictions of neurophysiological hypotheses, namely of multi-temporal resolution models as the “asymmetric sampling in time” model (Poeppel, 2003), using behavioral paradigms with filtered speech (e.g., Chait et al., 2015; Goswami et al., 2016). For example, Goswami et al. (2016) used low-pass filtered speech (at <4 Hz) in a speech perception behavioral task to support the hypothesis that dyslexic children have poor neural synchronization to slow speech amplitude modulations. The authors showed impoverished perception of low-passed filtered speech (i.e., speech where only prosodic information was available) that they interpreted as behavioral evidence for deficits in neural speech sampling in dyslexia.

## Materials and Methods

### Participants

Forty children (17 males; mean age 9 years 3 months, *SD* = 20.6 months) were recruited for the experiment in a school in Donostia-San Sebastián (Basque Country, Spain). The first group consisted of 20 children attending the 2nd grade (9 males; mean age 7 years 7 months, *SD* = 2.2 months). The second group consisted of 20 children attending the 5th grade (8 males; mean age 11 years, *SD* = 4.0 months). Four of the children in the group of 5th graders were left-handed.

Donostia-San Sebastián is a bilingual community where both Basque and Spanish coexist as official languages, and therefore parents were asked to fill out a questionnaire about the linguistic profile of their children. Following the analysis of the responses to the questionnaire, all children were classified as bilingual of Spanish and Basque. We used a measure of general exposure to the specific languages (in %) to determine if the children were Basque or Spanish dominant. Children with percentage of exposure to a language (Basque or Spanish) of more than 70% were considered dominant in that language. This analysis resulted in five children in the group of 2nd graders and one child in the group of 5th graders who were Basque-dominant. None of the children was diagnosed with any hearing disability, language-based or developmental disorder of any other kind, and it was stated that they suffered no ear infections at the time of testing. All children were recruited from a private school in Donostia-San Sebastián and tested under signed parental consent.

### Speech Perception Task

#### Stimuli

##### Target pseudowords

Target items were pseudowords with a low-density lexical neighborhood to avoid the influence of lexical information and require the participants to rely more strongly on sublexical phonological processing (Peelle and Davis, 2012; Peelle et al., 2013). The neighborhood density was calculated using the Levenshtein distance calculator of Yarkoni et al. (2008; mean neighborhood density 8.84).

To avoid any effects on target recall performance that could be due to the repetition of specific phonological and acoustic parameters across targets, we controlled for their length, stress pattern and phonemic structure. The target pseudowords were two- and three-syllable long items, and all syllables followed a consonant-vowel pattern (CVCV or CVCVCV). Half of the items (*n* = 40) were stressed in the first syllable and the other half (*n* = 40) in the last syllable. We counterbalanced the number of voiced and unvoiced consonants and vowels to avoid confounding variables due to sound complexity (difference in rise time, duration, etc.). Repetition of phonemes within targets was avoided. Finally for the properties of the pseudowords, only high-frequency bigrams were used (see Edwards et al., 2004 for manipulation of bigram frequency and its interaction with vocabulary size). Mean bigram frequency of the syllables was calculated with the tool *Espal* and was found to be overall high (Sample *Mean* = 7656, *SD* = 3948; database *Mean* = 5179; Duchon et al., 2013). The pseudoword was always preceded by a definite article, with gender counterbalanced across stimuli (*el* o *la*), and it was embedded in a Spanish sentence as the direct object of the verb (see Carrier Sentences for the different constituents composing the sentence).

##### Carrier sentences

Twenty different sentences were created. Sentences consisted of six to eight words (*M* = 7.0 words, *SD* = 0.6) and varied from 1.97 to 3.45 s (*M* = 2.8 s, *SD* = 0.3) in duration including four grammatical constituents. They were recorded by a female native speaker of Spanish in a sound-proof recording room. The sentences were not subject to digital manipulation, and the speaker read the pseudoword naturally within the sentence. The reader was blind to the purpose of the experiment and was instructed to read at a comfortable pace with a normal prosody. The sample frequency was fixed at 44.1 kHz.

Four grammatical constituents composed each sentence: (i) a subject phrase including a human agent whose gender was counterbalanced across sentences (e.g., *la abuela [the grandmother]*), (ii) a time complement which consisted of a high-frequency temporal adverb (e.g., *ayer [yesterday]*) or adverbial phrase (e.g., *por la mañana [in the morning]*), (iii) a high frequency transitive verb (log count *M* = 4.1, *MED* = 4.0, *SD* = 0.4) conjugated in the past simple, (iv) an object phrase which head was the target pseudoword (e.g., *la befu*, see Target Pseudowords for further details).

In order to avoid expectancy effects (i.e., children paying attention only to the target pseudoword, since the task was only to recall this element), we placed the target pseudoword in four different positions across our carrier sentences. Word order is highly flexible in Spanish, and this manipulation resulted in four different Spanish legal grammatical sentence structures (see **Table 1** for details on word order and time of appearance of the pseudoword within the carrier sentences).

**Table 1:** Word order and mean time of appearance of the Target (pseudoword) within the carrier sentences (P, position).

	Position of constituents within the sentence				Mean time appearance of the Target in ms (*SD*)
Target in P1	**Target**	Verb	Subject	Complement	188 (44.5)
Target in P2	Complement	**Target**	Verb	Subject	907 (168.3)
Target in P3	Subject	Verb	**Target**	Complement	1283 (137.5)
Target in P4	Complement	Subject	Verb	**Target**	2039 (211.7)

##### Primes

The primes were created via Matlab© (Mathworks, Natick, MA, United States) following the procedure for noise vocoding described in Peelle and Davis (2012). The audio signal from each sentence was divided into four logarithmic spaced channels. We decided to divide the signal into four channels to obtain an unintelligible, although still not excessively unpleasant sound for the children. The spectrum of each channel was extracted and a Hilbert transform was applied to extract the amplitude envelope of each signal. The signals were low-passed filtered at 8 Hz and used to modulate white noise in the same frequency bands. Finally, the channels were recombined to create the final amplitude envelope signal. The result was a modulated white noise which contained minimal phonemic information and matched the prosodic and syllabic amplitude envelope and the duration of the original sentence (see **Figure 1**). The prime was placed before the sentence with inter-stimulus interval (ISI) of 0 ms, since our intention was to create a continuous stimulus and gaps would encourage the discontinuity of attentional deployment.

For the null-prime conditions, we created a stream of random white noise that matched the original sentence in duration but contained no amplitude envelope information (steady white noise). The null-prime was also placed before the sentence with an ISI of 0 ms.

##### Noise: 6-talker babble

The babble-noise was constructed following the procedure used in Dole et al. (2012). Six speakers (three males, three females) were recorded in a soundproof room whilst reading passages of Spanish newspapers. From these individual six recordings, silences of more than 1 s were removed. Fragments containing pronunciation errors or exaggerated prosody were also discarded. We then applied a noise reduction to eliminate artifact interference for each of the six individual tracks, and, finally, mixed the tracks to create the 6-talker babble. The babble noise stimuli were then superimposed to half of the carrier sentences (*n* = 80) using Matlab© scripts at a SNR of 7 dB.

#### Procedure

Participants were sat in a quiet dim-lit room and listened to the sentences one by one through headphones (Sennheiser) and delivered via the Presentation© software. Stimuli were normalized at 80 dB and presented binaurally. The children were explicitly instructed to listen to the whole auditory stimulus (prime + sentence), but they were not informed about the aim of the experiment or the role of the prime. After the sentence, the experimenter asked a specific question that required the child to recall and produce the target pseudoword (e.g., Sentence: *Ayer la* ***befu*** *la tiró la madre [Yesterday the mother dropped the* ***befu****]”;* Question: *>Qué tiró la madre ayer?* [*What did the mother drop yesterday?*]; Response: *la* ***befu***). Participants could respond at their own pace and the next sentence was played only after the response was given. Responses were recorded by a mobile recording device (Zoom H2) and coded by the experimenter.

The same 20 carrier sentences were repeated across the four prime^∗^noise conditions in a random order. In that way, all carrier sentences were presented in a prime and null-prime condition within each listening condition (quiet and noise). Importantly, although the carrier sentences were the same across conditions, participants never listened to the same target pseudoword twice to avoid repetition effects on target perception and recall (total of 80 pseudowords). To avoid effects of the intrinsic characteristics of pseudowords (i.e., recall performance due to the characteristics of the pseudowords and not its time frame of appearance), two subgroups were created within each age group. The two subgroups listened to the same pseudowords and to the same carrier sentences, but recombined in a different way, i.e., pseudowords were not embedded in the same carrier sentences for both groups, and hence they listened to the target pseudowords in different conditions.

Eight practice sentences were presented prior to the experimental task. Breaks were made when necessary. The total duration of the experiment was approximately 15 min.

### Neuropsychological Screening

#### Non-verbal IQ

Non-verbal IQ was measured with the Matrix reasoning task of the WISC-IV. Direct scores were converted into scalar scores.

#### Phonological and Reading Tasks

##### Pseudoword repetition

This type of task has been extensively used to measure phonological short-term memory (e.g., Dollaghan and Campbell, 1998; Lallier et al., 2012). Participants were asked to repeat pseudowords varying in length (3 items of 2 syllables, 3 of 3 syllables, 3 of 4 syllables, and 3 of 5 syllables; total of 12). The syllable structure always followed a consonant-vowel (CV) pattern. The items were delivered through headphones one by one, and the children were asked to recall them. The final score corresponded to the total number of correct responses produced by the child (maximum = 12).

##### Phonemic deletion

This type of task has been extensively used to measure phonemic awareness (e.g., Stanovich et al., 1984). After a pseudoword was delivered through headphones, the participants were asked to repeat the entire item. Then, they had to repeat the item again but this time they were asked to omit the first sound. The task consisted of a total of 12 bi-syllabic items delivered in a random order (e.g., /timu/→/imu/). The final score corresponded to the total number of correct responses produced by the child (maximum score deletion based on the repetition of the child = 12).

##### Text reading

Participants were asked to read a short passage from the novel “The little prince” (“El principito,” written by Saint Exupéry) translated into Spanish (taken from Lallier et al., 2014) and consisting of 103 words and 9 lines. Participants were instructed to read as fast as possible minimizing the number of errors. Time (in seconds) and number of errors were recorded. Number of errors and total reading time were calculated as two separated scores.

##### Single word and pseudoword reading

Participants read single words (*n* = 30) and pseudowords (*n* = 30) taken from PROLEC-R (battery of evaluation of reading processes in Spanish; Cuetos et al., 2007). Participants were instructed to read as fast and as well as possible. Time and number of errors were recorded. Number of errors and total reading time were calculated as two separate scores.

## Data Analysis

To examine between-group differences in non-verbal IQ, reading and phonological skills, two-tailed *t*-tests were computed with grade as between-subject factor and Matrices (scalar scores), reading skills (word reading time, word reading errors, pseudoword reading time, pseudoword reading errors), and phonological skills (phoneme deletion scores, pseudoword repetition scores) as dependent variables.

For the speech perception task, the analysis was carried out fitting linear mixed-effect models (Baayen et al., 2008) with accuracy of target production (i.e., correct repetition of the whole target) as a dependent variable, and grade (two levels: Grades 2 and 5), listening condition (two levels: quiet and noise) and prime (two levels: null-prime and prime) as predictors. The complexity of the random-effects structure was increased in a series of models (cf. Barr et al., 2013), leading to a maximal converging model that contained all random effects that improved significantly the residual variance captured by the model. The *lme4* package (Bates et al., 2013) contained in R (R Development Core Team, 2011) was used to perform the mixed-effects models analysis. For analysis of accuracy measures, this package uses the inverse link function defined in the GLM family, through which the linear predictor is related to the conditional mean of the response. The output is hence expressed in *logits*. For clarity reasons, in the text we report inverse transformation from *logits*.

The random effects structure included items and subjects, and we added an additional random effect for target positions (1, 2, 3, 4). Although the four different sentence structures were grammatically legal, Spanish is a predominantly SVO language, and hence we expected that position of the target pseudoword could affect pseudoword recall. Regarding the interactions between the different fixed effects, we followed the same procedure, i.e., starting from the simplest model with no interactions and adding interactions that improved the fit of the data.

Before the recall scores obtained on the speech perception task were submitted to statistical analyses, all the oral responses of the children were double-checked by another native speaker of Spanish who was blind to the experimental conditions. The original coding of the experimenter and the verification coding matched for 95% of the items. A final decision for the items in conflict was reached upon agreement of both judges.

To perform correlation analyses, we calculated the benefit that the children obtained from the prime on pseudoword recall by subtracting the mean recall accuracy in the null-prime condition from the mean recall accuracy in the prime condition in both listening conditions separately (i.e., in quiet and in noise). Two-tailed partial correlations between the prime benefit values and reading as well as phonological skills were computed for both grades separately controlling for non-verbal IQ and chronological age.

## Results

### Neuropsychological Screening

Regarding text reading time [*t*(37) = 5.39; *p* < 0.001], 5th graders were significantly faster (*M* = 51.3 s; *SD* = 16.9) than 2nd graders (*M* = 102.4 s; *SD* = 38.7; see **Table 2**). No other difference between the two grades was found on non-verbal IQ, phoneme deletion, pseudoword repetition, text reading errors, word reading time, word reading errors, pseudoword reading time and pseudoword reading errors (*p*s > 0.05; see **Table 2**). Four of the children in Grade 5 were left-handed. We did not perform additional analyses to rule out the possibility that this could be a contributing factor to the group differences we reported, since hand-dominance does not seem to play a significant role in the distribution of cerebral asymmetries for processing temporal information of acoustic signals (Abrams et al., 2009). Language dominance (Basque vs. Spanish) did not correlate with any of the measures assessed in this study.

**Table 2:** Neuropsychological screening.

	Grade 2 (*n* = 19) *M* (*SD*)	Grade 5 (*n* = 20) *M* (*SD*)	Group difference *t*-values (2-tailed), *p*-values	
Chronological age (months)	91.9 (2.2)	132 (4)	-39.2	<0.001
Non-verbal IQ (WISC matrix subtest)	13.05 (3.1)	11.7 (2.5)	1.6	0.13
Reading skills				
Word reading – Number of errors	0.5 (0.77)	1.2 (1.5)	-1.7	0.1
Word reading – Time (s)	30.7 (12.93)	35.7 (10.6)	-1.33	0.2
Pseudoword reading – Number of errors	1.8 (1.80)	1.4 (1.2)	1.00	0.3
Pseudoword reading –Time (s)	50.4 (17.02)	53.6 (15.7)	-0.6	0.6
Text reading – Number of errors	2.4 (2.09)	2.3 (2.0)	0.2	0.9
Text reading – Time (s)	102.4 (38.7)	51.3 (16.9)	5.4	<0.001
Phonological skills				
Pseudoword repetition – Accuracy/12	10.2 (1.4)	10.4 (1.3)	-0.6	0.6
Phonemic deletion – Accuracy/12	9.2 (2.5)	8.7 (2.0)	0.9	0.4

### Speech Perception Task

Average accuracy of the two groups of children through the different conditions of the speech perception task is detailed in **Tables 3**, **4**.

**Table 3:** Mean accuracy (standard deviation in parenthesis) per Grade and Priming condition, averaged across participants for the Speech perception task.

	Grade 2 (*n* = 20)	Grade 5 (*n* = 20)
Null-Prime	0.51 (0.50)	0.62 (0.49)
Prime	0.52 (0.50)	0.72 (0.45)

**Table 4:** Mean accuracy (standard deviation in parenthesis) per Grade and Listening condition, averaged across participants for the Speech perception task.

	Grade 2 (*n* = 20)	Grade 5 (*n* = 20)
Quiet	0.76 (0.43)	0.91 (0.29)
Noise	0.27 (0.44)	0.43 (0.49)

As mentioned above, the complexity of the model was increased gradually following the procedure described in Barr et al. (2013). Specifically, a first model that included random intercept for Subject was successively compared to subsequent mixed-effect models containing by-Subject, by-Item and by-Position random intercepts. Random slopes for the different effects were added thereafter to allow the slope of the fixed effects (Grade, Prime, and Listening condition) to fluctuate across subjects, items and positions. The limitation of the estimation algorithm led to a simplification of the random effect structure with a maximal converging model that included random intercepts by-Subject (*SD* = 0.67), by-Item (*SD* = 0.76) and by-Position (*SD* = 0.64), and random slopes by-Subject for the effect of Prime (*SD* = 0.63) and by-Position for the effect of Listening condition (*SD* = 0.63). Results of the likelihood ratio test and changes in the coefficients of the model were used to check if including additional random effects improved the fit of the model. For each comparison, we report the intercept, the estimated regression coefficients (Estimate, β), the standard errors (SE), *z*- and *p*-values resulting from the mixed-effect model analysis.

A smaller probability of successful recall of the target pseudoword was found for the Noise (mean = 0.34, *SD* = 0.23) as compared to the Quiet (mean: 0.84; *SD*: 0.14) listening condition (β = 0.92; *SE* = 0.62; Wald’s *z* = 5.19; *p* < 0.001). Regarding the predictor Grade, the probability of successful answer was greater for the 5th graders (mean = 0.67, *SD* = 0.22) as compared to the 2nd graders (mean = 0.52, *SD* = 0.25), but this difference was only marginally significant (β = 0.62; *SE* = 0.57; Wald’s *z* = 1.81; *p* = 0.07). No main effect of prime was found in the maximally converging model (Null-prime mean = 0.56; *SD* = 0.25; Prime mean = 0.63; *SD* = 0.24; β = 0.47; *SE* = 0.60; Wald’s *z* = -0.30; *p* = 0.77).

**FIGURE 2:**
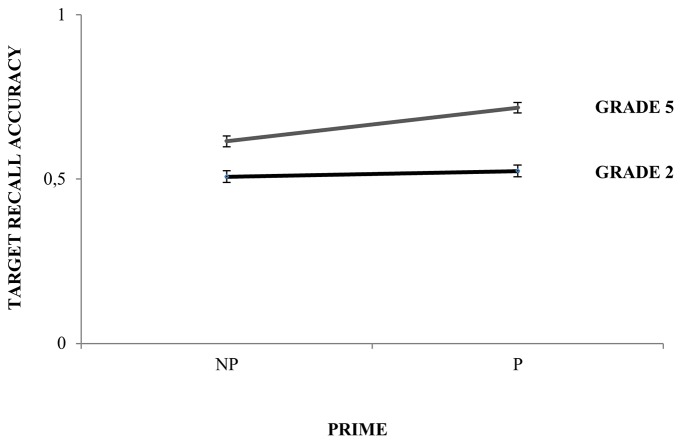
Per Grade performance on the speech perception task as a function of Prime, with target recall accuracy in *y*-axis and priming condition in *x*-axis (NP, null-prime; P, prime).

Regarding the interactions of the main predictors, the analysis revealed that Grade and Prime interacted significantly (β = 0.68; *SE* = 0.56; Wald’s *z* = 2.96; *p* < 0.01), such that 5th graders (Null-prime mean = 0.62, Prime mean = 0.72) obtained a larger benefit from the prime as compared to 2nd graders (Null-prime mean = 0.51; Prime mean = 0.52; see **Figure 2** and **Table 3**). The interaction Listening condition by Grade was also significant (β = 0.63; *SE* = 0.55; Wald’s *z* = 2.60; *p* < 0.01), revealing that performance was specially degraded in noise for the 2nd graders (Quiet mean = 0.76; Noise mean = 0.27) as compared to the 5th graders (Quiet mean = 0.91; Noise mean = 0.43; see **Table 4**).

Lastly for the fixed effects, the analysis revealed no significant Prime by Listening condition interaction (β = 0.67; *SE* = 0.64; Wald’s *z* = 1.26; *p* = 0.21).

**FIGURE 3:**
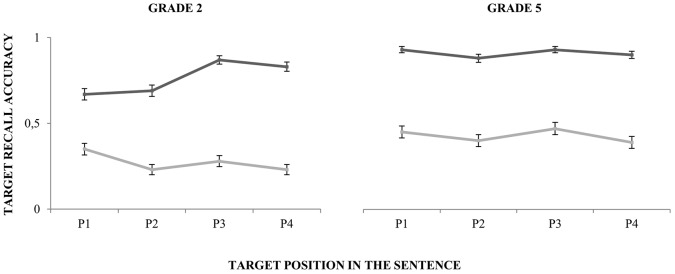
Per grade performance averaged across subjects across positions (P, position). Dark gray lines depict performance in the Quiet listening condition and light gray lines performance in the Noise listening condition.

In light of the Listening condition by Grade interaction, we performed a follow-up analysis to examine the possibility that the significant by-Position random slope for the effect of Grade was driven by one of the groups only. While the slope and intercept for the 5th graders remained considerably constant across Listening conditions and Positions, the inclusion of position as a random-effect in the final model seemed to be reflecting only the performance pattern of the 2nd graders (see **Figure 3** and **Table 5**).

**Table 5:** Mean accuracy (standard deviation in parenthesis) per Grade averaged across participants in the different positions and listening conditions of the Speech perception task.

	Grade 2 (*n* = 20)	Grade 5 (*n* = 20)
Quiet		
Position 1	0.67 (0.47)	0.93 (0.26)
Position 2	0.69 (0.46)	0.88 (0.33)
Position 3	0.87 (0.34)	0.93 (0.27)
Position 4	0.83 (0.38)	0.90 (0.30)
Noise		
Position 1	0.35 (0.48)	0.45 (0.50)
Position 2	0.23 (0.42)	0.40 (0.49)
Position 3	0.28 (0.45)	0.47 (0.50)
Position 4	0.23 (0.42)	0.39 (0.49)

### Correlation Analyses

One child from Grade 2 was removed from the correlation analyses, since he had not completed some of the phonological and reading tasks.

**FIGURE 4:**
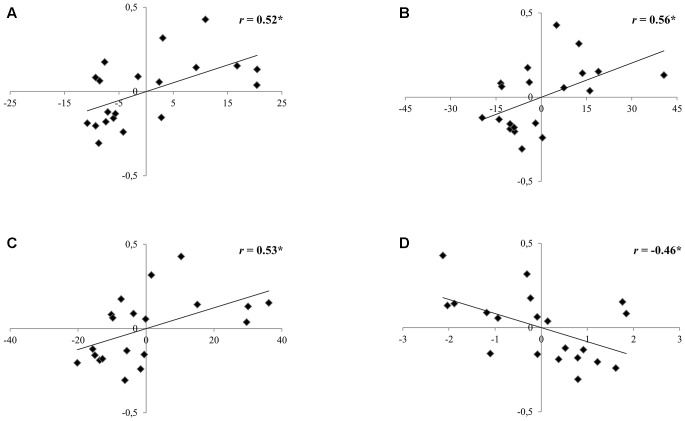
Scatterplots depicting the partial correlations between the recall benefit obtained from the prime in noise (in a scale from 0 to 1; *y*-axes) and **(A)** pseudoword reading time, **(B)** word reading time, **(C)** text reading time (in seconds; *x*-axes) and **(D)** pseudoword repetition scores (number of correct responses; *x*-axes) controlling for age and non-verbal IQ for the group of children in Grade 5. ^∗^*p* < 0.05.

Within the group of the 2nd graders, no correlation was found between performance on the speech perception task and neither reading nor phonological skills (see **Table 6**). Regarding the 5th graders, a negative correlation between pseudoword repetition scores and the benefit obtained from the prime in the noisy conditions was found (*r* = -0.46, *p* < 0.05), showing that the poorer the pseudoword repetition scores, the larger the prime benefit to recall the pseudoword presented in a noisy listening condition (**Figure 4**). Moreover, the slower the reading, the higher the benefit from the prime to recall pseudowords presented in a noisy listening condition (text reading: *r* = 0.53, *p* = 0.02; word reading: *r* = 0.56, *p* = 0.01; pseudoword reading: *r* = 0.52, *p* = 0.02; see **Figure 4**).

**Table 6:** *R*-values for two-tailed partial correlations between phonological and reading tasks and the benefit obtained from the prime in quiet and in noise controlling for non-verbal IQ and chronological age.

	Grade 2		Grade 5	
Benefit from the prime	In noise	In quiet	In noise	In quiet
Reading skills				
Word reading – Number of errors	-0.37	-0.40	-0.07	0.28
Word reading – Time (s)	-0.7	-0.17	0.56^∗^	-0.11
Pseudoword reading – Number of errors	-0.15	-0.29	0.37	-0.29
Pseudoword reading –Time (s)	-0.30	-0.10	0.52^∗^	-0.10
Text reading – Number of errors	0.01	0.19	0.09	-0.15
Text reading – Time (s)	-0.06	-0.23	0.53^∗^	-0.04
Phonological skills				
Pseudoword repetition – Accuracy/12	-0.08	0.02	-0.46^∗^	0.30
Phonemic deletion – Accuracy/12	-0.34	-0.32	0.06	0.15

*PW, pseudoword. ^∗^*p* < 0.05*.

## Discussion

The present study assessed whether spoken language comprehension of typically developing school-aged children can be enhanced by priming sentences with their own slow amplitude envelope only, in both quiet and noisy listening conditions. By priming sentences with their amplitude envelope low-pass filtered at 8 Hz (i.e., the speech amplitude envelope information was repeated across the prime and target sentence), we highlighted the impact of tracking the slow rhythmic structure of speech at prosodic and syllabic rates on speech intelligibility in Grade 5 children. In general, we observed that the processing of the sentences that were primed with their own speech envelope, as opposed to the sentences that were primed with non-modulated white noise (null-prime condition), were improved in children – as reflected by their better performance in pseudoword recall. We suggest that the speech amplitude envelope primes (without phonetic information) enhanced the processing of the speech amplitude envelope in the target sentences (via repetition of the speech amplitude envelope information), and this enhanced speech amplitude envelope processing enabled easier processing of the phonetic and lexical information in the target sentences. This study adds up to accumulating behavioral evidence (e.g., Chait et al., 2015; Goswami et al., 2016) supporting the predictions of multitemporal resolution neural models of speech (Zatorre and Belin, 2001; Boemio et al., 2005; Giraud et al., 2007; Obleser et al., 2008; Ghitza, 2011; Giraud and Poeppel, 2012; Luo and Poeppel, 2012; Saoud et al., 2012).

As predicted, our results revealed a main effect of listening condition, such that performance in pseudoword recall decreased significantly in the presence of the babble-noise. Interestingly, the prime led to a benefit in pseudoword recall across the quiet and the noise listening conditions similarly (absence of Listening condition by Prime interaction). However, the benefit from the prime seemed to be modulated by the age of the children. Group differences were observed such that the prime was overall more effective for the 5th graders than the 2nd graders. Nevertheless, we cannot discard that the effect of the prime for Grade 2 children was obscured by the high variability of this group’s performance, especially in the quiet listening condition (Quiet listening condition: Grade 2 *SD* = 0.43; Grade 5 *SD* = 0.29; Noise listening condition: Grade 2 *SD* = 0.44; Grade 5 *SD* = 0.49; see **Table 4**). Indeed, when considering individual prime benefits, eight out of the 20 children within Grade 2 showed an average effect of the prime greater than 5% across environments (12 children showed an effect of the prime larger than 5% in quiet). Hence, it could be that the variability in individual differences on the prime benefit could reflect different stages of development, which might be related to reading acquisition (see section *Relation between reading and entrainment to slow speech oscillations*). Related to this point, non-word repetition has been tightly related to vocabulary size (Edwards et al., 2004) and differences in vocabulary size could explain part of the differences between our groups. However, our paradigm was not designed to disentangle phonological short-term memory and vocabulary effects, and further research is needed to shed light on this relation.

Although our results suggest that the primes enhanced the auditory entrainment to the temporal structure of speech in the target sentence, which in turn enhanced its intelligibility, the current design does not allow us to distinguish among the following interpretations of the prime effect: (1) speech processing was enhanced by the pure repetition of the speech envelope; (2) the prime induced entrainment which was itself enough to improve the processing of the target sentence; (3) or both repetition and entrainment are responsible for the target intelligibility enhancement. In our opinion, the third interpretation is the most likely. Furthermore, replicating these results with electrophysiological methods is necessary. Regarding the interpretations (2) and (3), and specifically in the case of our group of 5th graders, the improvement of pseudoword recall by the presence of a prime could be providing further support to the idea that entrainment to the slow amplitude envelope of speech is important for speech intelligibility (Peelle and Davis, 2012; Peelle et al., 2013). Our behavioral results are in line with the predictions of multi-temporal resolution models (Poeppel, 2003; Ghitza, 2011; Giraud and Poeppel, 2012): entrainment to the slow oscillations of speech (by means of priming) could be supported through oscillatory mechanisms at low frequency bands (delta and theta bands), and this could in turn signal “acoustic landmarks” in which to anchor fast phonemic processing (gamma band). Moreover, the benefit obtained from the prime in the 5th graders in the noisy listening environment corroborates the hypothesis that the prime helped the children to selectively attend the target sentence, which facilitated phonemic information processing. Our results are in line with the neurophysiological hypothesis suggesting that the brain relies on acoustic information at low frequencies to segregate a target signal from a background noise and create a clearer neural representation of this signal (Zion Golumbic et al., 2012; Ding and Simon, 2014). However, Grade 2 children benefited less clearly from the prime in both the quiet and the noisy environments. In noisy listening situations in particular, this absence of main prime effect could stem from the overall poor performance of Grade 2 children. Moreover, the Grade by Listening condition interaction suggests that pseudoword recall performance was especially degraded by the babble noise for children in Grade 2 as compared to children in Grade 5. According to studies showing that noise affects the timing of neural responses at high frequency bands such as those recorded at the brainstem (Anderson et al., 2010), severe temporal neural disruptions may have been associated with poor speech in noise perception in the younger age group. Therefore, the babble noise must have strongly prevented Grade 2 children to encode pseudoword phonemic content. These difficulties may reflect problems faced by young children to integrate speech information across various temporal scales (prosodic-syllabic and phonemic) in noisy listening conditions.

Another factor that could have contributed to group differences was the position of the target pseudoword in the sentence. A follow-up analysis revealed that while the performance of 5th graders was stable in the different positions across listening conditions, position had a strong effect on performance for the 2nd graders, especially with regard to positions 1 and 2 when compared to positions 3 and 4 (see **Table 5** and **Figure 3**). On the one hand, this effect could be due to the fact that non-canonical orders of the sentence (pseudoword in positions 1 and 2) involved more complex language structures that younger children were not able to process fully automatically. On the other hand, group differences in the temporal deployment of attention might be at the base of this result. The 5th graders outperformed their younger peers when the target appeared in the initial positions of the sentence, supporting the idea that younger children might need more time to rapidly trigger auditory engagement to speech. An interesting follow-up of these results would be to further manipulate the time of appearance of the target pseudoword to explore the time course of attentional deployment over speech, which, at the neural level, could be reflecting the alternation between attentional engagement and disengagement moments and low- and high-excitability moments of neural tissue (Schroeder and Lakatos, 2009; Calderone et al., 2014; Lallier et al., 2017).

Lastly, it is important to note children in our speech perception task were instructed to repeat pseudowords. Although non-word repetition is a classical and extensively used task to measure phonological short term memory in children (e.g., Dollaghan and Campbell, 1998), it is a complex task involving auditory, phonological and speech-motor output processes (Gathercole, 2006). Therefore, one could ask whether the prime effects observed were the consequence of a boost all of these components rather than just speech intelligibility. Nevertheless, the main aim of the study was to test if a purely perceptual auditory prime would boost speech perception, and further studies are needed to disentangle the effect of auditory primes on phonological, auditory and motor output skills separately.

### Relation between Reading and Entrainment to Slow Speech Oscillations

Across grades, different correlation patterns were found between speech perception task performance, reading scores, and phonological scores. While no correlation was found within the 2nd graders group, phonological and reading measures correlated within the group of 5th graders. Moreover, a negative correlation was present between pseudoword repetition scores and the benefit obtained from the prime in the noisy condition, illustrating that children who were worse at repeating pseudowords benefited more from the prime that presumably facilitated the recall of the target pseudoword. Moreover, the slower readers among the 5th graders were those obtaining a greater benefit from the prime in noisy listening conditions. This result goes in line with recent theories of dyslexia (“temporal sampling framework,” Goswami, 2011) such that low reading abilities could be related to deficient sensitivity to slow speech amplitude modulations. In particular, children with good reading skills received a smaller benefit from the prime, most likely because the repetition of the amplitude envelope was redundant. On the contrary, the mere repetition of the frequencies of speech below 8 Hz improved speech intelligibility for children with less sensitivity to amplitude changes, who also were the ones with lower reading skills.

Importantly, these correlations were found for speech perception in the noisy conditions only. This is in line with reports on significant links between speech perception in noise and reading (Wible et al., 2002; Bradlow et al., 2003; Ziegler et al., 2009; Boets et al., 2011), while such link between reading and speech perception has not been consistently found in quiet listening settings (Brady et al., 1983; Bradlow et al., 2003). In fact, since fast (phonemic) and slow (syllabic and prosodic) information is fully available in quiet listening conditions, a few processing resources may be sufficient to overcome and hide the auditory deficits of poorer readers, at least behaviourally, which might manifest if the processing load (i.e., added noise) to perform the task increases (Soroli et al., 2010).

## Conclusion

The present results showed that prior availability of the amplitude envelope of speech (i.e., via priming) at prosodic and syllabic rates (below 8 Hz) enhances speech intelligibility, and that this effect is modulated by chronological age. Although our behavioral results are in line with the predictions of multitemporal resolution models of speech, this relation remains speculative; more evidence is needed to corroborate the idea that oscillatory entrainment is causally related to behavior, since the possibility that this activity is a mere by-product of other underlying processes remains open. Importantly, we observed that, only in the case of the older children (when the gap between children with different reading skills should start to be really meaningful), performance on the speech perception task significantly contributed to reading skills, such as the poorer the reading, the higher the prime benefit for processing speech in noisy listening conditions.

## Ethics Statement

This study was carried out in accordance with the recommendations of BCBL Ethics Committee with written informed consent from all subjects, parents, or tutors. All subjects gave written informed consent in accordance with the Declaration of Helsinki. The protocol was approved by the BCBL Ethics Committee.

## Author Contributions

PR-L, MaL, and MM wrote the manuscript. PR-L conducted the experiment and analyzed the data. PR-L and MiL prepared the stimuli.

## Conflict of Interest Statement

The authors declare that the research was conducted in the absence of any commercial or financial relationships that could be construed as a potential conflict of interest.
